# Route of Administration Affects Corticosteroid Sensitivity of a Combined Ovalbumin and Lipopolysaccharide Model of Asthma Exacerbation in Guinea Pigs

**DOI:** 10.1124/jpet.117.241927

**Published:** 2017-08

**Authors:** Alexander P. P. Lowe, Rhian S. Thomas, Anthony T. Nials, Emma J. Kidd, Kenneth J. Broadley, William R. Ford

**Affiliations:** Cardiff School of Pharmacy and Pharmaceutical Sciences, Cardiff University, Redwood Building, Cardiff (A.P.P.L., R.S.T., E.J.K., K.J.B., W.R.F.), and Discovery Biology, Respiratory Centre of Excellence for Drug Discovery, GlaxoSmithKline Medicines Research Centre, Stevenage (A.T.N.), United Kingdom

## Abstract

Lipopolysaccharide (LPS) contributes to asthma exacerbations and development of inhaled corticosteroid insensitivity. Complete resistance to systemic corticosteroids is rare, and most patients lie on a continuum of steroid responsiveness. This study aimed to examine the sensitivity of combined ovalbumin- (Ova) and LPS-induced functional and inflammatory responses to inhaled and systemic corticosteroid in conscious guinea pigs to test the hypothesis that the route of administration affects sensitivity. Guinea pigs were sensitized to Ova and challenged with inhaled Ova alone or combined with LPS. Airway function was determined by measuring specific airway conductance via whole-body plethysmography. Airway hyper-responsiveness to histamine was determined before and 24 hours post-Ova challenge. Airway inflammation and underlying mechanisms were determined from bronchoalveolar lavage cell counts and lung tissue cytokines. Vehicle or dexamethasone was administered by once-daily i.p. injection (5, 10, or 20 mg/kg) or twice-daily inhalation (4 or 20 mg/ml) for 6 days before Ova challenge or Ova with LPS. LPS exacerbated Ova-induced responses, elongating early asthmatic responses (EAR), prolonging histamine bronchoconstriction, and further elevating airway inflammation. Intraperitoneal dexamethasone (20 mg/kg) significantly reduced the elongated EAR and airway inflammation but not the increased bronchoconstriction to histamine. In contrast, inhaled dexamethasone (20 mg/ml), which inhibited responses to Ova alone, did not significantly reduce functional and inflammatory responses to combined Ova and LPS. Combined Ova and LPS–induced functional and inflammatory responses are insensitive to inhaled, but they are only partially sensitive to systemic, dexamethasone. This finding suggests that the route of corticosteroid administration may be important in determining corticosteroid sensitivity of asthmatic responses.

## Introduction

Corticosteroids are a mainstay of asthma treatment owing to their ability to reduce the late asthmatic response (LAR), airway hyper-responsiveness (AHR), and inflammatory responses to allergens (Palmqvist et al., 2005). Inhaled corticosteroids are used in regular maintenance therapy, whereas systemically administered corticosteroids are used in patients not achieving symptomatic control with standard treatments or experiencing an exacerbation (Boulet et al., 2015). The mechanism underlying the decreased responsiveness to inhaled corticosteroids in these patients is unknown, but several studies have indicated that lipopolysaccharide (LPS) contributes to both asthma exacerbations and the development of corticosteroid insensitivity (Goleva et al., 2008, 2013).

It is estimated that 5% of patients with asthma are corticosteroid-insensitive, but full resistance is rare, and most patients lie on a spectrum of responsiveness (Szefler et al., 2002; Bossley et al., 2009). In patients who are insensitive to inhaled corticosteroids, systemic corticosteroids, usually given orally, are often still effective (Ten Brinke et al., 2004; Hodgson et al., 2012). The continued effectiveness of oral corticosteroids may be due to suppression of inflammatory cells in the blood and bone marrow. In the bone marrow, corticosteroids suppress the maturation of hemopoietic cells, including eosinophils, an important cell type recruited to the airways in allergic asthma (Mao et al., 2004; Ben et al., 2008). Additionally, inflammatory cells, such as neutrophils, an important cell type in asthma exacerbations, are more corticosteroid-sensitive in the systemic circulation than in the lungs (Plumb et al., 2012). Inhaled corticosteroids can also access the systemic circulation and suppress inflammatory cell maturation in the bone marrow, depending on the dose and pharmacokinetics of the corticosteroid (Wood et al., 1999; Martin et al., 2002; Shen et al., 2002); however, access to the systemic circulation appears to decrease with increasing severity of asthma, thereby restricting the distribution of inhaled corticosteroids to the airways (Wood et al., 1999; Brutsche et al., 2000; Harrison and Tattersfield, 2003). Therefore, the tissue distribution of a corticosteroid may be an important determinant of the extent of inflammatory suppression.

Our previous study demonstrated that combining ovalbumin (Ova) with LPS challenge in sensitized guinea pigs increased the inflammatory and functional responses to allergen (Lowe et al., 2015). Moreover, it rendered these responses insensitive to doses of inhaled fluticasone propionate (FP) that were effective against functional and inflammatory responses to Ova alone (Lowe et al., 2015). A previous study by Komlósi et al. (2006) suggested that LPS decreases, but does not abolish, the sensitivity of Ova-induced inflammatory responses to i.p. dexamethasone; however, this study used a different combination of Ova and LPS and animal species (mice), making it difficult to generalize the findings to the guinea pig model we have developed. To our knowledge, no previous study has directly compared the sensitivity of allergen-induced functional and inflammatory responses to systemic and inhaled corticosteroids in a model of asthma or asthma exacerbation. Therefore, in the present study, we extend our earlier studies demonstrating insensitivity to inhaled fluticasone by examining the hypothesis that the functional and inflammatory responses induced by combining Ova and LPS demonstrate different sensitivities to inhaled and systemic dexamethasone. Ova and LPS are triggers for the inflammatory responses of the adaptive and innate immune systems, respectively. To identify possible underlying mechanisms for the steroid insensitivity and any differences with route of administration, we examined the lung levels of the cytokines interleukin (IL) IL-8, IL-13, and IL-17. IL-8 and IL-17 are released from macrophages and Th2 lymphocyte, respectively, and are involved in neutrophil chemotaxis (Ciepiela et al., 2015). IL-13 is released from macrophages and is involved in airway hyper-reactivity (Cockcroft and Davis, 2006).

## Materials and Methods

### 

#### Study Design.

**Fig. 1.**
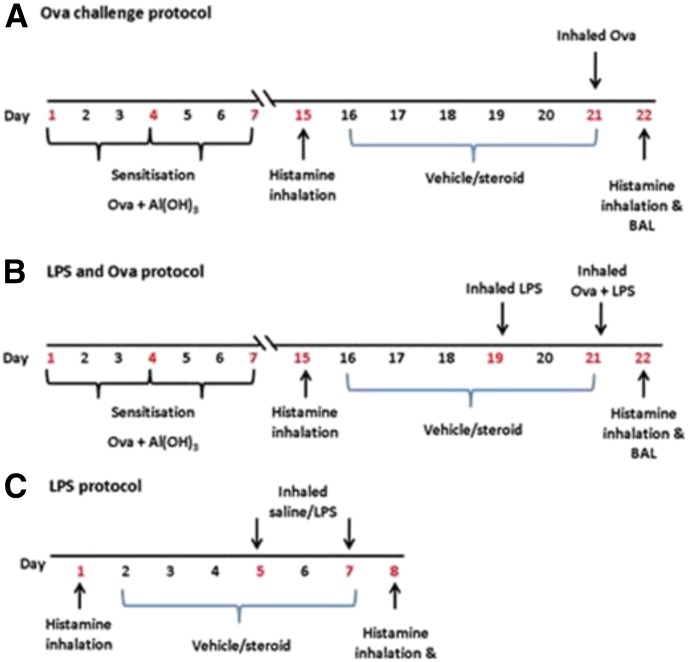
Protocols for Ova and LPS challenge: (A) Ova challenge, (B) Ova + LPS cochallenge, and (C) LPS challenge of naïve guinea pigs.

Guinea pigs (male, Dunkin-Hartley, 200–300 g; Charles River, Munich, Germany) were sensitized by i.p. injection of Ova (150 *µ*g) and Al(OH)_3_ (100 mg) in saline (1 ml) on days 1, 4, and 7 of the Ova protocols. They were challenged with inhaled ovalbumin (300 *µ*g/ml) or saline on day 21 in groups of six held in a Perspex exposure chamber (38 × 20 × 20 cm) using a DeVilbiss nebulizer at 0.3 ml/min and pressure of 20 psi (Fig. 1A). LPS (30 *µ*g/ml) or saline inhalation challenge was performed 48 hours pre-Ova and coadministered with Ova challenge (Fig. 1B). Nonsensitized guinea pigs were also exposed to LPS (30 *μ*g/ml) or saline on days 5 and 7 (Fig. 1C). Procedures were performed according to the Animals Act of 1986 (Scientific Procedures) and underwent ethical review by Cardiff University Biologic Standards Committee. The design and execution of the study followed Animal Research Reporting of In Vivo Experiments guidelines (McGrath et al., 2010).

#### Airway Function Measurement.

Airway function was measured as specific airway conductance (sGaw) in conscious, spontaneously breathing guinea pigs by plethysmography (Buxco Systems, DSI, St. Paul, MN). Lung function was recorded after final Ova, LPS, or saline challenge at intervals of 12 hours. Airways responses to aerosolized histamine were determined before and 24 hours after Ova or the final LPS/saline challenge using whole-body plethysmography. Histamine (0.3 mM, 2 minutes) was delivered by the use of a Buxco nebulizer chamber at 0.5 liters/minute and 10% duty (percentage every 6 seconds of nebulizing) per chamber. Histamine was selected to measure airways reactivity because it caused dose-related bronchoconstriction (0.1, 0.3, 0.5, and 0.9 mM) (unpublished data) in guinea pigs in our hands in naïve guinea pigs, the selected dose (0.3 mM) evoking minimal bronchoconstriction, which allowed for increases to be observed when there is hyper-reactivity. Lung function was measured before histamine inhalation and at 0, 5, and 10 minutes afterward.

#### Bronchoalveolar Lavage.

After the final histamine challenge, guinea pigs were sacrificed by an overdose of sodium pentobarbitone (Euthatal, 400 mg/kg, i.p.; Merial, Harlow, UK). Subsequently, bronchoaleveolar lavage of the whole lung was performed using saline (1 ml/100 g of guinea pig weight) instilled through a polypropylene cannula, inserted into the trachea, and left for 3 minutes. This process was repeated, and samples were pooled. Total cell numbers per milliliter were determined using a Neubauer hemocytometer. Leukocytes subpopulations of eosinophils, macrophages, neutrophils, and lymphocytes were determined from undiluted lavage fluid smears on glass microscope slides produced by a Shandon cytospin and subsequent staining with 1.5% Leishman’s solution in 100% methanol for 6 minutes. A minimum of 200 leukocyte cells were counted. Airway edema was determined by measuring lavage fluid protein content by BCA protein assay per the manufacturer’s instructions (Pierce Protein Biology, Fisher Scientific, Loughborough UK). Levels of IL-8, IL-13, and IL-17 were measured by enzyme-linked immunosorbent assay (R&D Systems, Abingdon, UK) using human antibodies on diluted homogenized lung samples. Human antibodies were used to prepare the standard curves. The use of human cytokine antibodies was selected for measuring guinea pig cytokines because at the time of performing these studies, the available guinea pig antibodies and kits were untried and unreliable. The human antibodies were effective in detecting and quantifying cytokines IL-8, IL-13, and IL-17 in guinea pig lung tissue, but there was no cross-reactivity with human IL-5, IL-12p70, interferon-*γ*, and IL-10, which were not therefore measured. Lung samples were prepared from 100 mg of right middle lung lobe using standard methods described previously (Thomas et al., 2011). Assays were carried out per the manufacturer’s instructions. The detection limits were 31 pg/ml for IL-8, 47 pg/ml for IL-13, and 16 pg/ml for IL-17. Cytokine levels were adjusted for total lung protein and expressed as picograms or nanograms per milligram of lung protein.

#### Drug Administration.

All chemicals were obtained from Sigma-Aldrich (Gillingham, UK) or Fisher-Scientific (Loughborough, UK) unless stated otherwise. Ovalbumin prepared from chicken eggs by ion exchange (VWR International Ltd, Leicestershire, UK) was dissolved in saline with Al(OH)_3_ in suspension and stirred for at least 2 hours before injection.

Dexamethasone or vehicle was administered either by i.p. injection or inhalation (Fig. 1). Vehicle [25% dimethylsulfoxide, 75% saline] or dexamethasone (5, 10, or 20 mg/kg) was administered by once-daily bilateral i.p. injection (0.5 ml each side). Inhaled vehicle (30% ethanol, 30% dimethylsulfoxide, 40% saline) or dexamethasone (4 or 20 mg/ml) was administered by nebulizer for 15 minutes twice daily into a Perspex whole-body exposure chamber (38 × 20 × 20 cm) exposing six animals at a time, using the same DeVilbiss nebulizer used for Ova challenge. The mass median aerodynamic diameter of the particles generated by this system was 2.79 ± 0.22 *µ*m. Drug administration took place from days 16 to 21 in Ova protocols and days 2 to 7 in LPS-only protocols, 30 minutes before any Ova or LPS administration.

#### Data Analysis.

Lung function data were plotted as a percentage of baseline sGaw. The EAR (0–6 hours) and LAR (6–12 hours) were expressed as the peak bronchoconstriction, displayed as a histogram next to the time course and as area under the curve to account for differences in the timing and duration of the allergen responses. The duration of the EAR was also analyzed as the time taken to return to 50% of peak EAR sGaw values. Results are plotted as the mean ± S.E.M.

Student’s *t* tests were used for the comparison of differences between two groups or data points. One-way analysis of variance (ANOVA) followed by Dunnett’s post-test was used when two or more groups were being compared with a control group or Bonferroni post-test when two or more groups were being compared with each other. A two-way ANOVA was not considered appropriate for comparisons between time courses as we were not interested in whether there were differences between responses at different times but between treatments. It would be too severe a test and likely to yield type 2 errors (no differences detected between treatments when there were clear differences at individual time points, such as the peaks of EAR and LAR). The nonparametric Kruskal-Wallis ANOVA or Mann–Whitney *U* tests were used where appropriate for data that did not display equal variances. No statistical comparisons were made between Ova + LPS and LPS alone because the experimental conditions were very different, LPS being examined in naïve unsensitized animals and Ova + LPS in sensitized animals. *P* values less than 0.05 were considered significant.

## Results

### Effect of LPS Challenge on Ova-Induced Functional and Inflammatory Responses

**Fig. 2.**
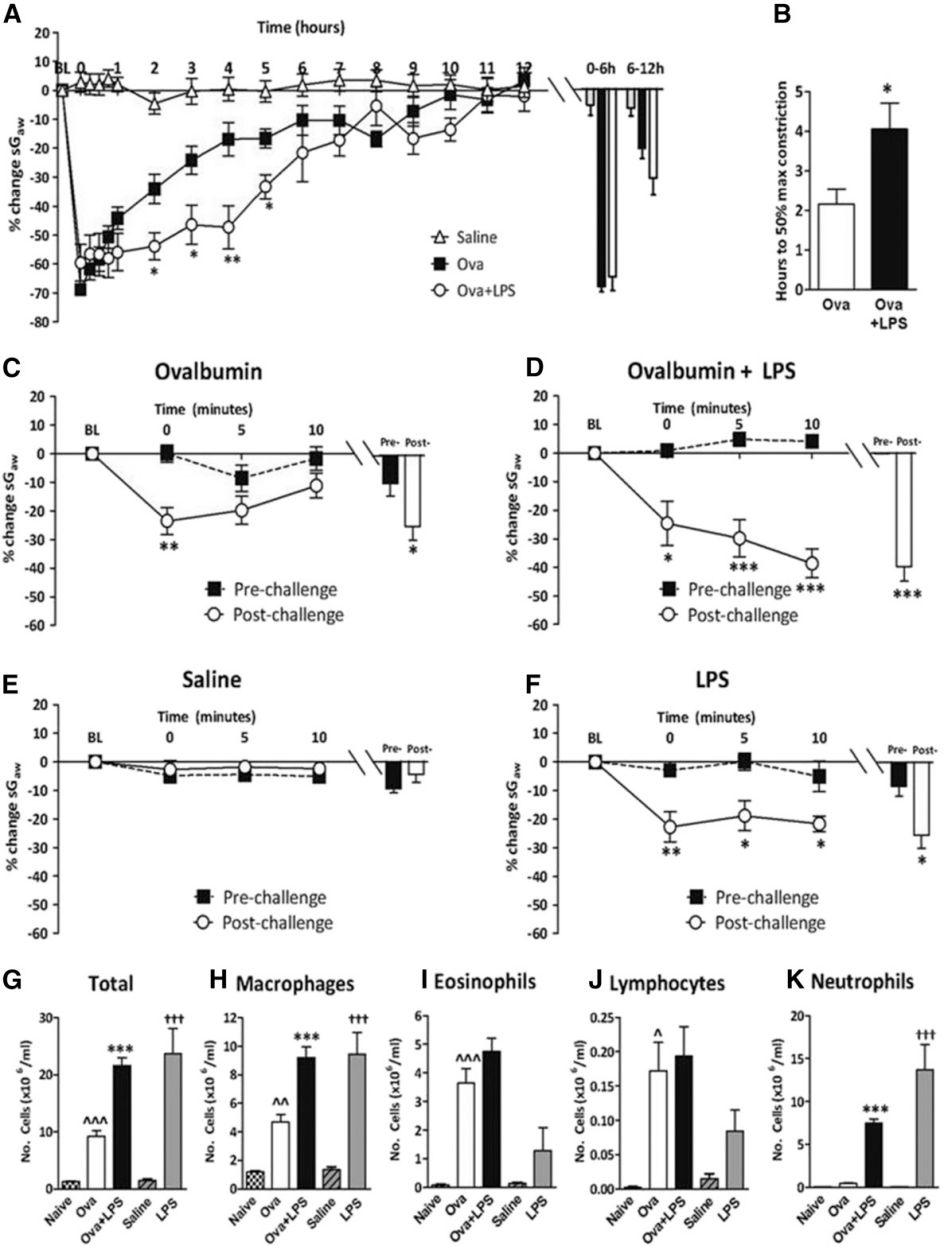
Guinea pigs were sensitized with Ova and challenged with Ova or saline alone or Ova + LPS, or unsensitized guinea pigs were challenged with LPS. (A) Mean time courses for changes in sGaw. Histograms represent the maximum bronchoconstriction values recorded during the EAR (0–6 hours) and LAR (6–12 hours); mean changes in sGaw are expressed as mean ± S.E.M. percent change from baseline before Ova challenge. (B) Analysis of the time taken for EAR to recover to 50% of peak bronchoconstriction values. (C and F) Response of the airways to nebulized histamine (0.3 mM, 2 minutes) 24 hours before and 24 hours after (C) Ova and (D) Ova + LPS challenge; (E) the second saline challenge; (F) the second LPS challenge. Histograms represent the mean peak response (G) total cell, (H) macrophage, (I) eosinophil, (J) lymphocyte, and (K) neutrophil counts in bronchoalveolar fluid. Naïve (unsensitized and unchallenged) groups are also shown for comparative purposes only; *n* = 6 or 7. *Significantly different from Ova challenge or prehistamine challenge where appropriate, *P* < 0.05; ** *P* < 0.01; ***P < 0.001; ^^significantly different from naïve, *P* < 0.01, ^^^ *P* < 0.001; †††Significantly different from saline, *P* < 0.001; performed with a two-tailed *t* test or one-way ANOVA, followed by Bonferroni or Kruskal-Wallis ANOVA post-test where appropriate.

**Fig. 3.**
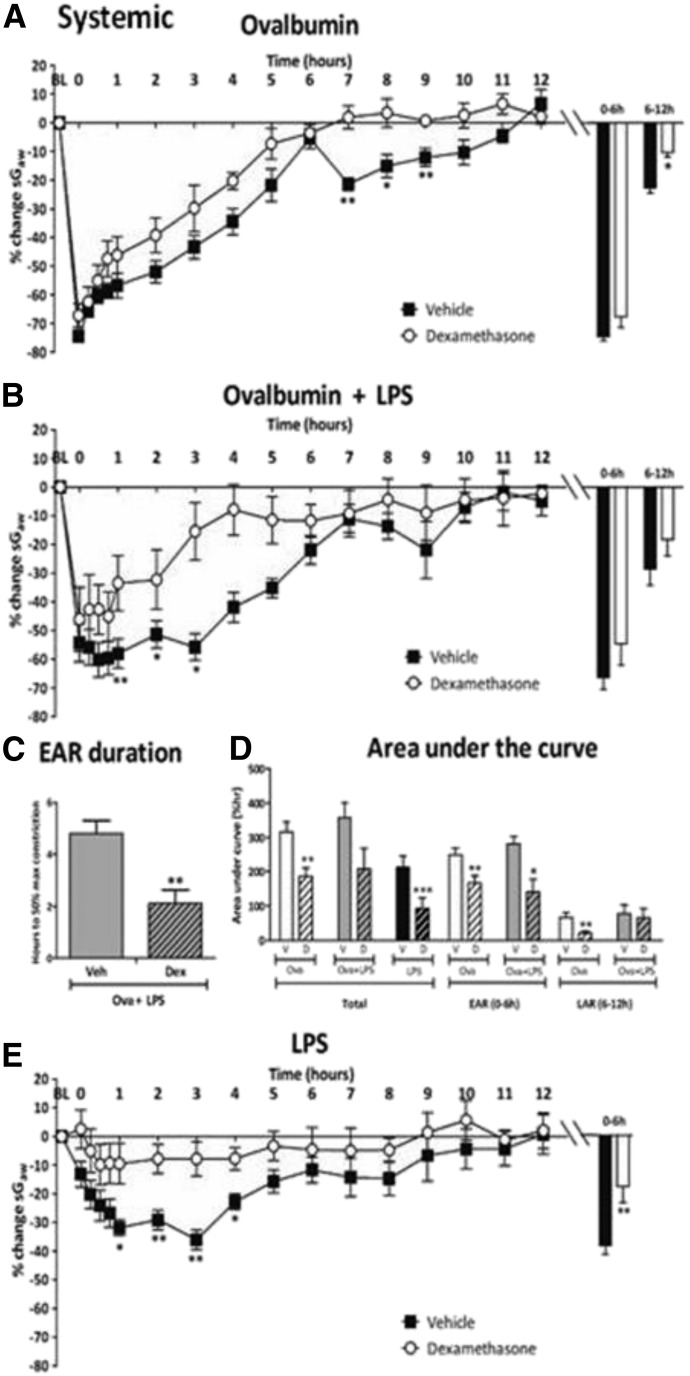
Guinea pigs were sensitized with ovalbumin (Ova) and challenged with (A) Ova alone or (B) Ova + LPS or unsensitized guinea pigs challenged with LPS and treated with once-daily i.p. vehicle or dexamethasone (20 mg/kg). (A and B) Mean time courses for changes in sGaw. Histograms represent the maximum bronchoconstriction values recorded during the EAR (0–6 hours) and LAR (6–12 hours); mean changes in sGaw are expressed as mean ± S.E.M. percent change from baseline before Ova challenge. (C) Analysis of the time taken for the EAR to recover to 50% of peak bronchoconstriction values. (D) AUC analysis of sGaw values for 0–12 hours, 0–6 hours, and 6–12 hours post-Ova challenge; also shown are data for naïve guinea pigs challenged with LPS; (E) mean time courses for changes in sGaw for naïve guinea pigs treated with LPS; *n* = 6 to 7; *Significantly different from vehicle treatment, *P* < 0.05; **P < 0.01; ***P < 0.001; performed with one-way ANOVA followed by Dunnett’s post-test.

Saline challenge produced no significant changes to the baseline sGaw over 12 hours (Fig. 2A). Ova challenge induced an immediate bronchoconstriction, characteristic of the EAR and a second bronchoconstriction at 9 to 10 hours indicative of a LAR (Fig. 2A). The addition of LPS to Ova challenge increased the duration of the EAR (Fig. 2A) and increased time to reach a 50% reduction in peak EAR sGaw (Fig. 2B). LPS alone induced a bronchoconstriction lasting 4 hours, the peak being significantly greater (−35.7% ± 2.7%) than with saline challenge (−19.5% ± 4.8%) (data not shown, but compare with vehicle-treated animals shown in Fig. 3E).

Ova challenge induced AHR, demonstrated by a significant increase in the immediate bronchoconstriction by histamine 24 hours after Ova challenge (Fig. 2C). The addition of LPS to Ova challenge altered the bronchoconstrictor response to histamine 24 hours after Ova inhalation, with continued, progressive, significant decreases in sGaw from 0 to 10 minutes after histamine challenge (Fig. 2D). Saline challenge did not significantly change the bronchoconstrictor response to histamine (Fig. 2E), but it was significantly increased 24 hours after challenge with LPS alone (Fig. 2F). When Ova challenge was compared with naïve guinea pigs, there were significant increases in total cells, macrophages, eosinophils, and lymphocytes (Fig. 2, G–J). IL-13 (8.7 ± 1.2 ng/mg vs. 3.4 ± 0.5 ng/mg) and IL-17 (113.5 ± 5.7 ng/mg vs. 41.9 ± 15.4 ng/mg) levels were also significantly increased after Ova challenge compared with naïve guinea pigs, respectively (data not shown). Lavage fluid protein levels and IL-8 levels were unchanged by Ova challenge (data not shown). The addition of LPS to Ova challenge further increased total cells, macrophages, and neutrophils compared with Ova alone (Fig. 2, G, H, and K). Lung IL-8 (38.6 ± 3.4 pg/mg vs. undetectable, respectively) and lavage fluid protein (4.2 ± 0.5 ng/mg vs. 0.4 ± 0.1 ng/mg, respectively) were also significantly increased with the addition of LPS to Ova (data not shown). Eosinophils (Fig. 2I) and cytokine IL-13 and IL-17 levels (data not shown) were unchanged by the addition of LPS to Ova challenge. LPS challenge alone significantly increased total cells, macrophages, and neutrophils compared with saline challenge (Fig. 2, G, H, and K). Lavage fluid protein (3.2 ± 0.3 ng/mg vs. 0.4 ± 0.1 ng/mg) and IL-8 (25.4 ± 3.3 pg/mg vs. undetectable) were also significantly increased compared with saline (data not shown).

#### Inhaled and Intraperitoneal Dexamethasone on Early and Late Asthmatic Responses.

##### Intraperitoneal dexamethasone.

Ova challenge in vehicle-treated guinea pigs induced an EAR and LAR, the latter being significantly reduced by i.p. dexamethasone, 20 mg/kg (Fig. 3A), but not by the 5- or 10-mg/kg doses (−21.4% ± 7.4%, −21.3% ± 2.0%, respectively) compared with vehicle (−22.6% ± 1.9%; data not shown). In Ova + LPS groups, i.p. dexamethasone (20 mg/kg) reduced the duration of the extended EAR, with a significant reduction in the bronchoconstriction between 1 and 3 hours after Ova challenge, but not the peak EAR or LAR (Fig. 3B). This result was revealed as a significantly reduced time to 50% recovery of the EAR (Fig. 3C). In the Ova-alone groups, the area-under-the-curve (AUC) analysis revealed significant reductions in the EAR and LAR with i.p. dexamethasone 20 mg/kg compared with vehicle. In Ova + LPS groups, i.p. dexamethasone (20 mg/kg) significantly reduced the EAR AUC, but not the LAR (Fig. 3D). LPS challenge alone in vehicle-treated animals elicited bronchoconstriction 0–4 hours postexposure, peaking at 3 hours (Fig. 3E), which along with AUC (Fig. 3D**)** was significantly attenuated by i.p. dexamethasone (20 mg/kg).

##### Inhaled dexamethasone.

**Fig. 4.**
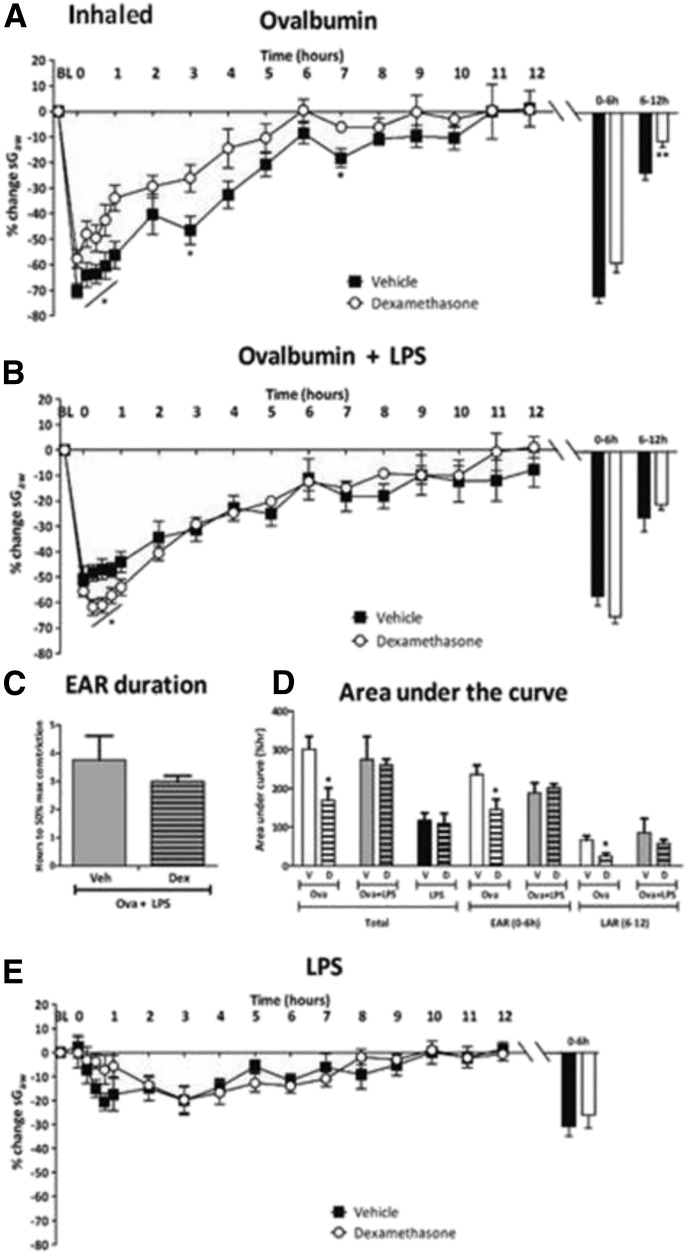
Guinea pigs were sensitized with Ova and challenged with (a) Ova alone or (B) Ova + LPS and treated with twice-daily inhaled vehicle or dexamethasone (20 mg/ml). Histograms represent the maximum bronchoconstriction values recorded during the EAR (0–6 hours) and LAR (6–12 hours); mean changes in sGaw are expressed as mean ± S.E.M. percent change from baseline before Ova challenge. (C) Analysis of time taken for the EAR to recover to 50% of peak bronchoconstriction values; (D) AUC analysis of sGaw values for 0–12 hours, 0–6 hours, and 6–12 hours post-Ova challenge; also shown are data for naïve guinea pigs challenged with LPS; *n* = 6 or 7. *Significantly different from vehicle treatment *P* < 0.05; **P < 0.01; ***P < 0.001; performed with one-way ANOVA followed by Dunnett’s post-test.

In Ova-alone groups, the peak EAR was significantly attenuated by inhaled dexamethasone (20 mg/ml) (−57.9% ± 3.6%) compared with vehicle (−70.8% ± 2.6%, respectively) Fig. 4A), but not by the lower dose (4 mg/ml) of inhaled dexamethasone (−73.2% ± 2.7%; data not shown). The peak LAR was also attenuated by inhaled dexamethasone (20 mg/ml) (−11.1% ± 2.0%) compared with vehicle (−21.3% ± 2.6%) (Fig. 4A) but not by inhaled dexamethasone, 4 mg/ml (−21.3% ± 2.6%; data not shown). In Ova + LPS groups, inhaled dexamethasone (20 mg/ml) significantly increased the bronchoconstriction 0–1 hour postchallenge (e.g., 15 minutes: −61.8% ± 3.1%) compared with vehicle (−48.3% ± 3.1%) (Fig. 4B) and the peak (63.7% ± 2.4% vs. −55.6% ± 3.6%, respectively), but it did not affect AUC or recovery of the EAR or the LAR (Fig. 4, C and D). In LPS only groups, the bronchoconstriction induced by LPS challenge was also insensitive to inhaled dexamethasone (20 mg/ml) (Fig. 4E). Inhaled vehicle reduced the peak bronchoconstriction to LPS compared with i.p. vehicle, suggesting inhibitory effects of daily inhaled vehicle upon airway function responses to LPS.

#### Inhaled and Intraperitoneal Dexamethasone on Airway Hyper-responsiveness.

##### Intraperitoneal dexamethasone.

**Fig. 5.**
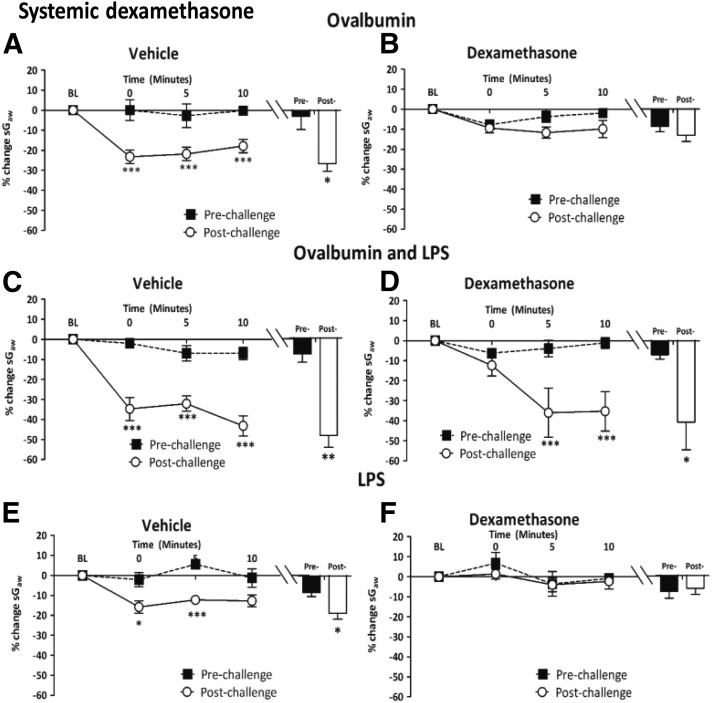
Responses of airways to nebulized histamine delivered in a plethysmograph (0.3 mM, 2 minutes) to guinea pigs (A and B) sensitized and challenged with Ova and treated with once-daily i.p. (A) vehicle or (B) dexamethasone (20 mg/kg); (C and D) sensitized and challenged with Ova + LPS treated with once-daily i.p. (C) vehicle or (D) dexamethasone (20 mg/kg); (E and F) challenged with LPS and treated with once-daily i.p. (E) vehicle or (F) dexamethasone, 20 mg/kg. Histograms represent the mean peak response to histamine before and after Ova, Ova + LPS, or LPS challenge. Mean changes in sGaw are expressed as mean ± S.E.M. percent change from baseline. A negative value represents a bronchoconstriction. *n* = 6 or 7. *Significantly different from vehicle treatment *P* < 0.05; **P < 0.01; ***P < 0.001; performed with a two-tailed *t* test.

Ova alone in i.p. vehicle-treated guinea pigs induced AHR as an increase in bronchoconstriction in response to histamine (Fig. 5A). This response was attenuated by i.p. dexamethasone, 10 mg/kg (postchallenge, −12.6% ± 3.7% vs. prechallenge, −12.0% ± 2.1%; data not shown) and 20 mg/kg (Fig. 5B), but not the 5 mg/kg dose (data not shown). In the Ova + LPS groups, the prolonged bronchoconstrictor response to histamine in vehicle-treated guinea pigs (Fig. 5C) was not significantly reduced by i.p. dexamethasone, 20 mg/kg (Fig. 5D). The increased bronchoconstrictor response after LPS challenge alone in vehicle-treated guinea pigs (Fig. 5E) was abolished by i.p. dexamethasone, 20 mg/kg (Fig. 5F).

**Fig. 6.**
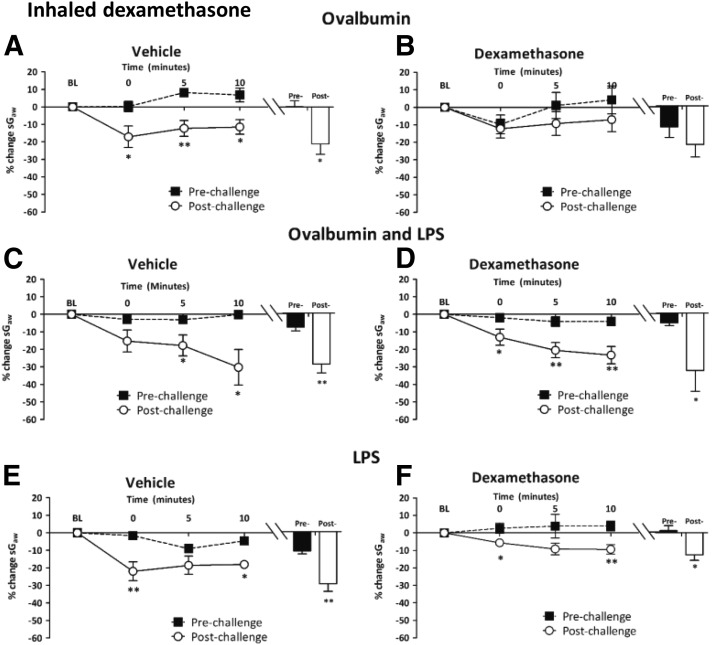
Responses of airways to nebulized histamine delivered in a plethysmograph (0.3 mM, 2 minutes) to guinea pigs (A and B) sensitized and challenged with Ova and treated with twice-daily inhaled (A) vehicle or (B) dexamethasone (20 mg/ml); (C and D) sensitized and challenged with Ova + LPS and treated with twice-daily inhaled (C) vehicle or (D) dexamethasone (20 mg/ml); (E and F) nonsensitized and challenged with LPS and treated with once-daily inhaled (E) vehicle or (F) dexamethasone 20 mg/ml. Histograms represent the mean peak response to histamine before and after Ova, Ova + LPS, or LPS challenge. Mean changes in sGaw are expressed as mean ± S.E.M. percent change from baseline. A negative value represents a bronchoconstriction. *n* = 6 or 7. *Significantly different from vehicle treatment, *P* < 0.05; **P < 0.01; ***P < 0.001; performed with a two-tailed *t* test.

*Inhaled dexamethasone.* In inhaled vehicle-treated guinea pigs, Ova alone increased the bronchoconstriction resulting from histamine (Fig. 6A). This increase was significantly attenuated with inhaled dexamethasone, 4 mg/ml (postchallenge, −15.5% ± 4.1% versus prechallenge, −10.1% ± 5.3%;)(data not shown) and 20 mg/ml (Fig. 6B). In the Ova + LPS groups, the significantly increased peak bronchoconstrictor response to histamine in inhaled vehicle-treated guinea pigs (Fig. 6C) was unchanged by inhaled dexamethasone (20 mg/ml) (Fig. 6D). Similarly, in the LPS-alone groups, the increased response to histamine in inhaled vehicle-treated guinea pigs (Fig. 6E) was not significantly attenuated by inhaled dexamethasone (20 mg/kg) (Fig. 6F).

#### Inhaled and Intraperitoneal Dexamethasone on Airway Inflammation.

##### Intraperitoneal dexamethasone.

**Fig. 7.**
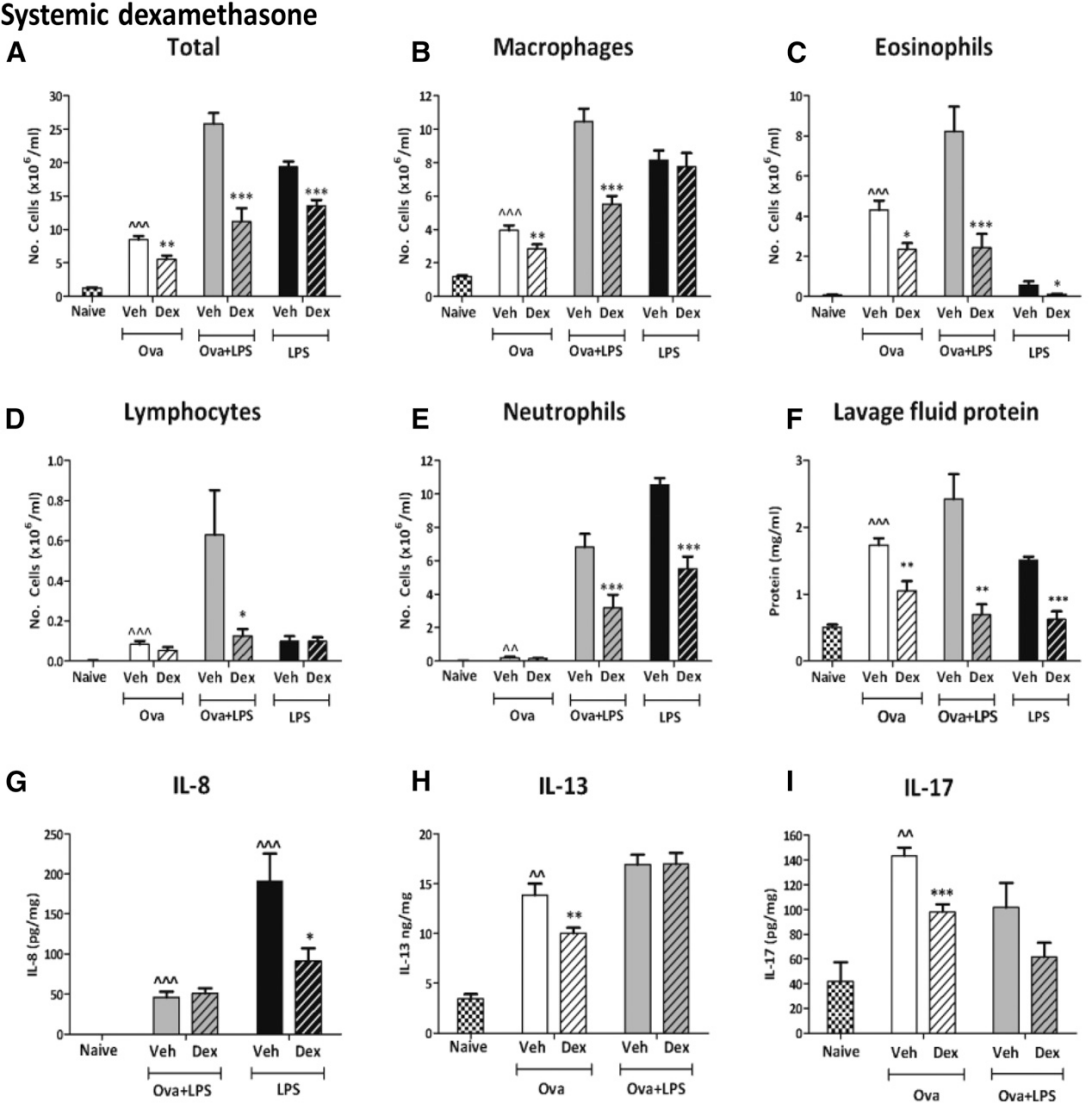
Total cell (A), macrophage (B), eosinophil (C), lymphocyte (D), and neutrophil (E) counts and lavage fluid protein levels (F) in the bronchoalveolar fluid and IL-8, (G), IL-13 (H), and IL-17 (I). Guinea pigs were sensitized and challenged with Ova, sensitized with Ova, and challenged with Ova + LPS or nonsensitized guinea pigs challenged with LPS. Guinea pigs were treated with once-daily i.p. vehicle or dexamethasone (20 mg/kg). Results are expressed as mean ± S.E.M.; *n* = 6 or 7; *Significantly different from vehicle treatment, *P* < 0.05; **P < 0.01; ***P < 0.001; **^***P* < 0.05; **^^**significantly different from naïve, *P* <0 .01; **^^^***P* < 0.001; performed with a two-tailed *t* test or Mann-Whitney *U* test where appropriate.

Ova challenge alone of vehicle-treated animals increased total cells and all individual cell types (Fig. 7, A–E**)**. It also increased total protein in lavage fluid and levels of the cytokines IL-13 and IL-17 (Fig. 7, H and I). Intraperitoneal dexamethasone (20 mg/kg) reduced total cells (Fig. 7A), macrophages **(**Fig. 7B), eosinophils (Fig. 7C), and lymphocytes (Fig. 7D**)** compared with vehicle. Neutrophils were not changed by any dose of dexamethasone (Fig. 7E). Lavage fluid protein was significantly reduced by i.p. dexamethasone, 10 mg/kg (1.2 ± 0.1 mg/ml; data not shown) and 20 mg/kg (Fig. 7F**),** but not 5 mg/kg, compared with vehicle (1.7 ± 0.2 mg/ml)(data not shown). IL-8 was not detectable in samples from naïve or Ova-challenged guinea pigs and is therefore not shown. Intraperitoneal dexamethasone (20 mg/kg) significantly decreased both IL-13 (Fig. 7H) and IL-17 (Fig. 7I) compared with vehicle treatment.

In Ova + LPS groups, i.p. dexamethasone (20 mg/kg) significantly reduced the total cells and all individual cell types compared with vehicle treatment (Fig. 7, A–E). Lavage fluid protein was also significantly reduced with i.p. dexamethasone (20 mg/kg) to nearly naïve levels compared with vehicle treatment (Fig. 7F). In contrast, IL-8, IL-13, and IL-17 were not significantly reduced by i.p. dexamethasone (20 mg/kg) (Fig. 7, G–I).

In the LPS-alone group, i.p. dexamethasone (2 mg/kg) significantly reduced total cells (Fig. 7A), eosinophils (Fig. 7C), and neutrophils (Fig. 7E) compared with vehicle treatment. Significant decreases in lavage fluid protein (Fig. 7F) and IL-8 (Fig. 7G) with i.p. dexamethasone (20 mg/kg) compared with vehicle treatment were also observed.

##### Inhaled dexamethasone.

**Fig. 8.**
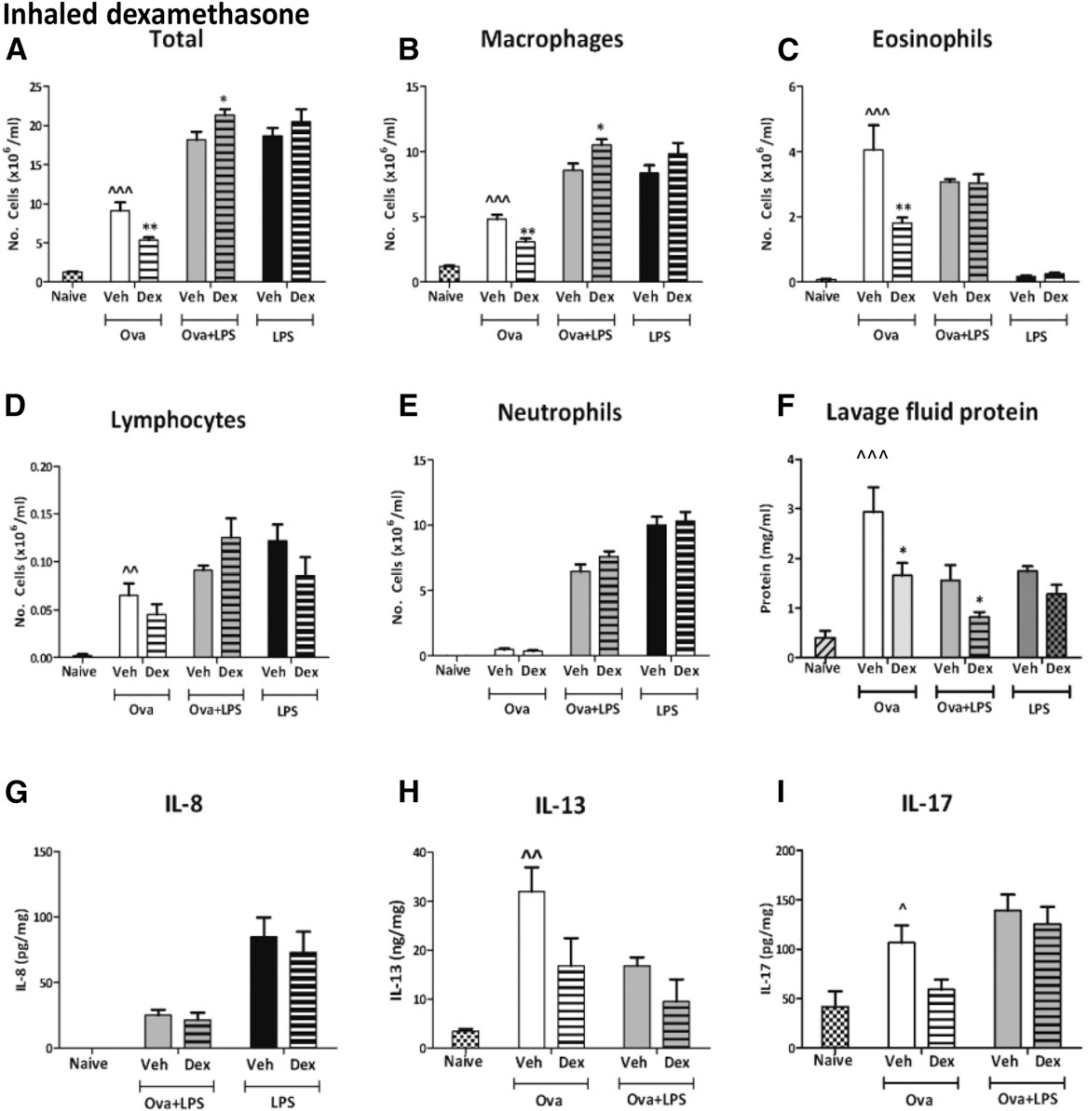
The total cell (A), macrophage (B), eosinophil (C), lymphocyte (D), and neutrophil (E) counts and lavage fluid protein levels (F) in the bronchoalveolar fluid and IL-8 (G), IL-13 (H), and IL-17 (I). Guinea pigs were sensitized and challenged with Ova, sensitized with Ova, and challenged with Ova + LPS or nonsensitized guinea pigs challenged with LPS. Guinea pigs were treated with twice-daily inhaled (C) vehicle or (D) dexamethasone (20 mg/ml). Naïve (unsensitized and challenged) guinea pigs are also shown for comparative purposes only. Results are expressed as mean ± S.E.M.; *n* = 6 or 7, *Significantly different from vehicle treatment, *P* < 0.05; **P < 0.01; ***P < 0.001; **^***P* < 0.05; **^^**significantly different from naïve, *P* < 0.01, **^^^***P* < 0.001; performed with a two-tailed *t* test or Mann-Whitney *U* test where appropriate.

Ova alone increased total cells, macrophages, eosinophils, lymphocytes, lavage fluid protein, IL-13, and IL-17 in vehicle-treated guinea pigs compared with naïve guinea pigs (Fig. 8, A–D, F, H, and I). Inhaled dexamethasone (4 mg/ml) reduced total cell numbers (6.1 ± 0.7 × 10^6^/ml) compared with vehicle treatment (9.1 ± 1.1 × 10^6^/ml) and eosinophils (2.1 ± 0.3 × 10^6^/ml) compared with vehicle treatment (4.1 ± 0.8 × 10^6^/ml; data not shown). Inhaled dexamethasone (20 mg/ml) also significantly reduced total cells (Fig. 8A) and eosinophils (Fig. 8C) compared with vehicle treatment. Additionally, inhaled dexamethasone (20 mg/ml) significantly reduced macrophages (Fig. 8B) and lavage fluid protein (Fig. 8F). IL-8 was not detectable after Ova alone and is therefore not shown. IL-13 and IL-17 were reduced by dexamethasone (20 mg/ml), but this decrease was not statistically significant (Fig. 8, H and I).

In the Ova + LPS groups, inhaled dexamethasone (20 mg/ml) did not significantly reduce any leukocytes or cytokines measured; however, inhaled dexamethasone (20 mg/ml) significantly increased total cell numbers compared with vehicle treatment (Fig. 8A), with a corresponding increase in macrophages (Fig. 8B). In contrast, lavage fluid protein was significantly reduced by inhaled dexamethasone (20 mg/ml) compared with vehicle (Fig. 8F). It is worth noting that in the vehicle-treated groups, the addition of LPS to Ova did not increase the numbers of eosinophils as seen in untreated animals (Fig. 2I) or in animals treated with i.p. vehicle (Fig. 7C). This was probably not due to inconsistency of the eosinophil response but to an effect of inhaled vehicle. Similarly, LPS alone induced pulmonary inflammation and lavage fluid protein, and IL-8 levels were unchanged with inhaled dexamethasone (20 mg/ml) (Fig. 8, A–G).

## Discussion

This study is the first to demonstrate directly that corticosteroid insensitivity varies according to the route of administration in an animal model of asthma exacerbation. It also demonstrated a divergence between functional responses, such as AHR, and inflammatory responses in their sensitivity to corticosteroids. This study may have important implications for the future treatment of corticosteroid-insensitive asthma and improve understanding of why oral corticosteroids are an effective rescue medication in patients who do not respond to high-dose inhaled corticosteroids.

Inhaled and systemic corticosteroids are effective at reducing allergen-induced inflammatory and functional responses in guinea pigs (Toward and Broadley, 2004; Evans et al., 2012; Ford et al., 2013). Dose-response assessment of dexamethasone on Ova-induced functional responses revealed that the highest doses of inhaled (20 mg/ml) and i.p. (20 mg/kg) dexamethasone reduced the LAR and AHR. The processes underlying functional responses are initiated during the EAR and mediated by inflammatory cell, including eosinophils, macrophages and lymphocytes, and cytokines, including IL-5 and IL-13 (Gauvreau et al., 2000; Toward and Broadley, 2004; Bradding et al., 2006). Correspondingly, these cell types were all reduced in Ova groups by the highest doses of dexamethasone. IL-13 and IL-17 levels were also reduced by dexamethasone, although statistically significant reductions were demonstrated only with i.p. dexamethasone. This dose was then used to examine the corticosteroid sensitivity of functional and inflammatory responses to combined Ova + LPS challenge. The addition of LPS to Ova challenge elongated the EAR, prolonged the bronchoconstrictor response to histamine, and increased airway inflammation, confirming the findings of our previous study (Lowe et al., 2015). Unlike with Ova challenge alone, functional and inflammatory responses to Ova + LPS were differentially sensitive to inhaled and i.p. dexamethasone. Airway inflammation and the prolonged EAR were decreased by i.p. dexamethasone, but AHR and LAR were not. By contrast, both functional and inflammatory responses were insensitive to inhaled dexamethasone, confirming our previous findings (Lowe et al., 2015) with FP in this model. The overall finding is that corticosteroid insensitivity in this model is dependent on the route of administration.

The EAR is mediated by basophils and mast cells and has been demonstrated to be insensitive to inhaled FP in humans and guinea pigs (Palmqvist et al., 2005; Evans et al., 2012; Lowe et al., 2015). In contrast, the present study shows that recovery of the EAR after Ova alone was reduced by dexamethasone administered by both the inhaled and the i.p. routes without any effect on the peak EAR. In the Ova + LPS group, however, the duration of the elongated EAR was reduced by i.p. dexamethasone but not by inhaled dexamethasone. This finding suggests that a component of the Ova alone–induced EAR is corticosteroid-sensitive and that the process that mediates the elongated EAR after Ova + LPS challenge is also sensitive to systemic corticosteroid. The sensitivity of Ova + LPS responses to dexamethasone corresponds with the sensitivity to dexamethasone of the bronchoconstriction induced by LPS alone, which was also reduced by i.p. but not inhaled dexamethasone. Correspondingly, neutrophils, but not macrophages, were reduced in the group given LPS alone, suggesting they may be a mediator of this response. This observation is supported by a study in mice, which also demonstrated an association between neutrophil depletion using vinblastine and a reduction in the bronchoconstriction to LPS (Lefort et al., 2001). Thus, temporal overlap between the LPS- and Ova-induced bronchoconstrictions may explain why the EAR in the Ova + LPS groups is partially sensitive to systemic corticosteroid. Furthermore, the insensitivity of the elongated EAR response in the Ova + LPS groups to inhaled dexamethasone suggests that the elongated EAR is mediated in part by factors external to the lung. Histamine and several other substances released from airway mast cells are generally considered mediators of the EAR. Therefore, decreased airway mast cell numbers or histamine release is unlikely to mediate the elongated EAR, as these cells are localized to the airways and would be expected to be equally sensitive to both routes of corticosteroid administration (Toward and Broadley, 2004; James et al., 2012).

Other functional responses, including AHR and the LAR, were rendered insensitive to both routes of dexamethasone administration with the addition of LPS to airways challenge with Ova. Therefore, these findings confirm that the corticosteroid insensitivity of the AHR and LAR after Ova + LPS is not specific to FP or the inhaled route of administration (Lowe et al., 2015). A previous study has also demonstrated a similar insensitivity of AHR to i.p. dexamethasone in Ova + LPS–challenged mice (Komlósi et al., 2006). As both the LAR and AHR have been demonstrated to be associated with airway inflammation (Gauvreau et al., 2000; Toward and Broadley, 2004; Dente et al., 2008), it is interesting that Komlósi et al. (2006) found that eosinophils, macrophages, and lymphocytes remained corticosteroid-sensitive; however, the absolute numbers of inflammatory cells remained high and similar to counts in Ova + vehicle groups. Both findings are supported in the current study and may indicate that the remaining inflammatory cell population is still sufficient and actively releasing mediators, such as interferon-*γ* and IL-13, that promote bronchoconstriction (Yang et al., 2009; Kinyanjui et al., 2013). Alternatively, free radicals generated from macrophages and neutrophils may produce epithelial damage, resulting in corticosteroid insensitive-AHR in the Ova + LPS groups. Epithelial damage disrupts the metabolism of bronchoconstrictive substances such as histamine, explaining the increased length of histamine-induced bronchoconstrictions (Hoshi et al., 1996; Haddad, 2004). Moreover, epithelial wound closure has been reported to be unaffected or even suppressed by corticosteroids, which would mean that AHR mediated by this mechanism would likely be corticosteroid-insensitive (Dorscheid et al., 2006) (http://www.ncbi.nlm.nih.gov/pmc/articles/PMC3615997/). Additionally, epithelial disruption may expose the underlying parasympathetic nerves and increase neural reflexes in the airways, promoting the LAR in rats and guinea pigs (Raemdonck et al., 2012; Smit et al., 2014).

The sensitivity of Ova + LPS-induced responses coincided with the sensitivity of LPS-induced responses to inhaled and systemic dexamethasone, suggesting insensitivity in the Ova + LPS model may be the result of intrinsic insensitivity after LPS. Consistent with previous studies, LPS responses were demonstrated to be insensitive to inhaled dexamethasone but sensitive to systemic dexamethasone (O’Leary et al., 1996; Trapp et al., 1998; Toward and Broadley, 2004); however, the lack of sensitivity to i.p. dexamethasone—such as AHR in Ova + LPS compared with LPS groups—suggests that the effect of LPS on Ova-induced responses is more than additive. Additionally, LPS + Ova challenge induced inhaled corticosteroid insensitivity of the allergen-driven responses such as eosinophilia, which is associated with allergen rather than LPS challenge.

LPS was administered by inhalation to the airways, likely resulting in corticosteroid insensitivity mainly localized to this tissue compartment. This result would limit the anti-inflammatory activity of inhaled corticosteroids, which are localized mainly in their distribution to the airways, particularly in severe asthma (Wood et al., 1999; Brutsche et al., 2000; Harrison and Tattersfield, 2003); however, a systemically administered corticosteroid is distributed to tissues beyond the lung including the blood and bone marrow, reducing leukocyte maturation and migration to the airways from these tissues (Mao et al., 2004; Ben et al., 2008). Thus, the compartmentalization of corticosteroid insensitivity may explain why lavage inflammatory cells are decreased by systemic, but not by inhaled, corticosteroid in the Ova + LPS groups.

Neutrophils develop in the bone marrow and are recruited to the airways from the systemic circulation (Wang and Arase, 2014). The sensitivity of neutrophils to corticosteroids differs between the systemic circulation and the lung. In the lung, neutrophils demonstrate decreased expression of glucocorticoid receptor-*α* compared with the blood and consequently are relatively corticosteroid-insensitive in the airways (Plumb et al., 2012).Therefore, i.p. dexamethasone, with a systemic distribution, may reduce neutrophil numbers in a tissue compartment where they are relatively sensitive to corticosteroids. In contrast, inhaled corticosteroids demonstrate low systemic distribution (Wood et al., 1999; Martin et al., 2002; Shen et al., 2002). Thus, in the current study, the reduction in neutrophils with systemic, but not inhaled, dexamethasone in Ova + LPS and LPS groups may be due to differences in corticosteroid distribution. The corticosteroid insensitivity of lung neutrophils may be further exacerbated in conditions of high oxidative stress as a result of reduced histone deacetylase transferase-2 activity and increased expression of glucocorticoid receptor-*α*, decreasing corticosteroid sensitivity (Hamilos et al., 2001; Ito et al., 2004; Mercado et al., 2011). Thus, the elevated neutrophil numbers present in Ova + LPS–challenged guinea pigs may represent a distinct corticosteroid-insensitive lung neutrophil population, supported by the continued elevation of lung IL-8 levels, which were not reduced by systemic dexamethasone (Trapp et al., 1998).

Corticosteroid insensitivity does not seem to extend to the increase in lavage fluid protein response to Ova + LPS, since levels were decreased by inhaled and systemic dexamethasone. This finding suggests that differences in the sensitivity of Ova + LPS responses to i.p. and inhaled dexamethasone are not due to drug delivery issues and would be unlikely to account for the large differences in anti-inflammatory action between these two routes of administration.

This study has demonstrated that the LAR, AHR, airway inflammation (eosinophils and macrophages), lung cytokines (IL-13 and IL-17), and lavage fluid protein levels induced by Ova alone are sensitive to inhaled and systemic dexamethasone. The addition of LPS to the Ova exposure abolished the effect of inhaled dexamethasone on these parameters. It also reduced the effectiveness of systemic dexamethasone on the LAR and AHR. Have our results with inflammatory cytokines thrown further insight into the site of steroid resistance? The generation of IL-8, IL-13, and IL-17 by Ova + LPS was resistant to both systemic and inhaled steroid; however, neutrophils were inhibited by systemic dexamethasone but not by the neutrophil chemoattractants IL-8 and IL-17. Therefore the site of steroid action on neutrophil migration must be independent of these cytokines, possibly on neutrophil survival through inhibition of their apoptosis (Meagher et al., 1996. The insensitivity of IL-13 and AHR to both routes of administration after Ova + LPS indicates that AHR resistance is linked to events above IL-13 generation in the inflammatory cascade possibly at the site of Th2 cells. These results suggest that the route of corticosteroid administration may be important in determining the sensitivity of asthmatic responses to these agents. These results reflect the clinical situation, where patients who are insensitive to inhaled corticosteroids frequently demonstrate some sensitivity to systemic corticosteroids. This suggests that the Ova + LPS model is clinically relevant and may facilitate better modeling of corticosteroid insensitivity in asthma, currently an area of unmet clinical need.

## Abbreviations

AHR: airway hyper-responsiveness
ANOVA: analysis of variance
AUC: area under the curve
EAR: early asthmatic response
FP: fluticasone propionate
IL: interleukin
LAR: late asthmatic response
LPS: lipopolysaccharide
Ova: ovalbumin
sGaw: specific airway conductance
